# Influence of Modified Fucoidan and Related Sulfated Oligosaccharides on Hematopoiesis in Cyclophosphamide-Induced Mice

**DOI:** 10.3390/md16090333

**Published:** 2018-09-13

**Authors:** Natalia Yu. Anisimova, Nadezhda E. Ustyuzhanina, Maria I. Bilan, Fedor V. Donenko, Natalia A. Ushakova, Anatolii I. Usov, Mikhail V. Kiselevskiy, Nikolay E. Nifantiev

**Affiliations:** 1 N.N. Blokhin National Medical Research Center of Oncology, Kashirskoe shosse, 24, 115478 Moscow, Russia; n_anisimova@list.ru (N.Y.A.); donenko.f20010@yandex.ru (F.V.D.); 2 N.D. Zelinsky Institute of Organic Chemistry, Russian Academy of Sciences, Leninsky Prospect 47, 119991 Moscow, Russia; bilan@ioc.ac.ru (M.I.B.); usov@ioc.ac.ru (A.I.U.); 3 V.N. Orekhovich Research Institute of Biomedical Chemistry, Pogodinskaya str. 10, 119121 Moscow, Russia; natalia.ushakova@ibmc.msk.ru

**Keywords:** granulocyte colony-stimulating factor, fucoidan, synthetic oligosaccharide, hematopoiesis, immunosuppression, cyclophosphamide

## Abstract

Immunosuppression derived after cytostatics application in cancer chemotherapy is considered as an adverse side effect that leads to deterioration of quality of life and risk of infectious diseases. A linear sulfated (1→3)-α-l-fucan **M-Fuc** prepared by chemical modification of a fucoidan isolated from the brown seaweed *Chordaria flagelliformis*, along with two structurally related synthetic sulfated oligosaccharides, were studied as stimulators of hematopoiesis on a model of cyclophosphamide immunosuppression in mice. Recombinant granulocyte colony-stimulating factor (**r G-CSF**), which is currently applied in medicine to treat low blood neutrophils, was used as a reference. Polysaccharide **M-Fuc** and sulfated difucoside **DS** did not demonstrate significant effect, while sulfated octasaccharide **OS** showed higher activity than **r G-CSF**, causing pronounced neutropoiesis stimulation. In addition, production of erythrocytes and platelets was enhanced after the octasaccharide administration. The assessment of populations of cells in blood and bone marrow of mice revealed the difference in mechanisms of action of **OS** and **r G-CSF**.

## 1. Introduction

Cytostatics application in cancer chemotherapy results in a number of side effects, including the suppression of different branches of hematopoiesis. Cyclophosphamide (**CPh**) is an alkylating agent that is currently used in the treatment of various forms of malignant neoplasms [1]. The main side effect of **CPh** is connected with suppression of rapidly proliferating hematopoietic stem/progenitor cells (HSPCs) and results in acute neutropenia, lymphopenia, erythropenia, and thrombocytopenia [2,3,4].

The functional activity of HSPCs has been shown to depend mainly on specific local microenvironment formed by bone marrow vascular niches [5,6]. Endothelium of bone marrow is known to express cell adhesion molecules involved in cell signaling by interaction with glycoprotein and glycolipid ligands of HSPCs [6,7,8]. Recently, it was shown that selectins and integrins play a crucial role in regulation of hematopoiesis in experimental animals with myelosuppression after chemotherapy [9,10].

Recombinant granulocyte colony-stimulating factor (**r G-CSF**) is currently applied in medicine to treat low blood neutrophils [11]. However, **r G-CSF** does not possess a significant stimulating effect on platelet and erythrocyte germs. Therefore, the development of an alternative drug that is able to stimulate several branches of the hematopoiesis is still a challenge.

**Figure 1:**
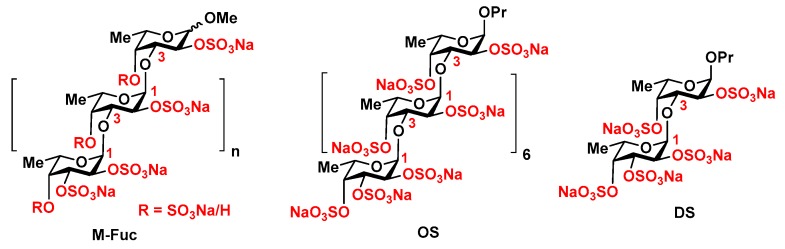
Modified fucoidan **M-Fuc** [14] and related synthetic octasaccharide O**S** [15,16] and disaccharide **DS** [17,18].

Recently, fucoidan from the brown seaweed *Chordaria flagelliformis* was shown to be an effective stimulator of hematopoiesis in a model of cyclophosphamide-induced mice [12]. This biopolymer demonstrated the level of activity comparable with that of **r G-CSF** regarding neutropoiesis stimulation. Additionally, this compound was found to be capable of stimulating erythropoiesis and thrombopoiesis. The main disadvantage of the native polysaccharide from *C. flagelliformis* is the very complex structure. The backbone composed of the repeating (1→3)-linked α-l-fucopyranosyl residues is decorated by numerous α-l-fucopyranosyl, α-d-glucuronyl, and more complex disaccharide branches [13]. Random sulfation of a backbone and branches significantly mask the regularity of the polysaccharide. To simplify the structure of this polysaccharide, several chemical transformations have been performed resulting in preparation of sulfated linear fucoidan **M-Fuc** with molecular weight ~5 kDa (Figure 1) [14].

In this paper, we report the study of a modified fucoidan **M-Fuc**, along with a related synthetic octasaccharide **OS** [15,16] and disaccharide **DS** [17,18] (Figure 1), as stimulators of hematopoiesis on a model of **CPh** immunosuppression in mice.

## 2. Results

Modified fucoidan **M-Fuc** is a linear polysaccharide composed of the (1→3)-linked α-l-fucopyranosyl residues bearing the sulfate groups at O-2 or at both O-2 and O-4 (Figure 1). In our study, it was prepared from the high molecular weight fucoidan from the brown seaweed *Chordaria flageliformis* by chemical elimination of branches, partial depolymerization, and sulfation [14]. The mean molecular weight of **M-Fuc** was estimated as ~5 kDa. The degree of sulfation of **M-Fuc** was 1.7. The synthetic per-*O*-sulfated linear *n*-propyl octa-(1→3)-α-l-fucoside **OS** [15,16] may be regarded as a fragment of **M-Fuc**. Its MW was ~2.8 kDa and the degree of sulfation was 2.0. The synthetic (1→3)-linked difucoside **DS** [17,18] with MW ~0.5 kDa and the degree of sulfation 2.0 could be considered as a low molecular weight analogue of **M-Fuc**.

The effect of compounds **M-Fuc**, **OS**, and **DS** on hematopoiesis was studied on a model of **CPh**-induced immunosuppression in mice. Recombinant **r G-CSF** (Leicyta) was applied as a reference. Intact animals were regarded as a positive control. Active concentrations of the compounds have been determined previously [12,19]. The values of the hematological parameters in various groups of mice are presented in Table 1. The levels of white and red blood cells (WBC, RBC), platelets, and hemoglobin were also determined.

These data showed that **CPh** injection led to a decrease in the concentration of leucocytes by 2.9 times, erythrocytes by 1.7 times, and hemoglobin by 1.5 times, while leading to an increase in the level of platelets by 1.4 times. The subsequent treatment with **r G-CSF** resulted in a tendency of normalization of the RBC, hemoglobin, and platelets levels. At the same time, the level of leukocytes in blood increased, but its median value did not reach the control level. Treatment with fucoidan **M-Fuc** and disaccharide **DS** did not produce significant changes in the hematologic parameters. The most pronounced effect—even exceeding that of **r G-CSF**—was observed in the presence of octasaccharide **OS**. In the **CPh** + **OS** group, the recovery of the WBC, RBC, and hemoglobin concentrations to the positive control level was observed. The level of platelets remained elevated.

An assessment of the populations of the white blood cells revealed that cyclophosphamide injection led to a decrease in levels of monocytes, lymphocytes, and neutrophils (Table 2). Neutrophils were the most sensitive to **CPh** impact (100 mg/kg, 1 × 4 days); their concentration in **CPh**-group was lower by 5 times compared to that in the intact control. After injection of **M-Fuc** or **DS** in a therapeutic regime, no significant recovery of the cells was observed, while administration of **r G-CSF** and **OS** led to a sufficient increase in the cell concentration. Notably, the effect of **OS** was more pronounced than that of **r G-CSF** in all cases of white blood cells populations.

Further elucidation of lymphocyte subpopulations revealed that after **CPh** treatment, a predominant depletion of the cells was observed due to the subpopulation of CD3^+^CD4^+^ cells, which was reflected by a decrease in the CD4^+^/CD8^+^ index from 1.94 ± 0.28 to 0.88 ± 0.10 (Table 3). Injection of **OS** recovered CD4^+^/CD8^+^ index up to a level of control group (1.93 ± 0.19), while the use of **r G-CSF** had a similar tendency but with lower efficiency (1.38 ± 0.05). Intergroup statistical analysis (vs. control and vs. CPh) showed no significant changes in NK content in the blood of animals of all groups.

As molecules of cell adhesion selectins and integrins are known to play a key role in immune response, their levels were measured on granulocytes (Table 4). After the course of **CPh**, an increase in CD11c^+^ and CD62p^+^ granulocytes by ~1.5 times was observed, indicating the circulation predominantly of highly differentiated activated “old” cells. Interestingly, after **r G-CSF** treatment, the levels of CD11c and CD62p expression were 2 times lower than those in the case of **OS**. It could be supposed that in the latter case, there was a release of a large number of granulocytes capable of immune response.

Next, the effect of the tested compounds on the functional activity of the blood granulocytes was studied (Table 5). It was found that the introduction of **CPh** did not lead to a decrease in the functional activity of blood granulocytes released in a high rate of bacterial capture. The suppression of the anti-infectious immunity of patients after the cytostatic treatment is connected with a decrease in the number of the effector cells rather than with their reactivity. In the **CPh** + **r G-CSF** group, a slight decrease in the rate of phagocytic activity compared to the intact control was observed, which was probably due to mobilization of youth cells from the bone marrow; however, there was still a high level of reactive oxygen species (ROS) generation. By contrast, in the **CPh** + **OS** group, an increase of phagocytic activity of the granulocytes was observed compared to a control group.

**Figure 2:**
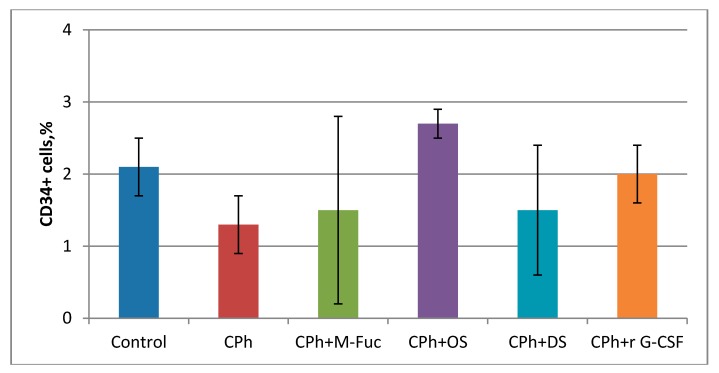
Content of progenitor cells CD34^+^ in bone marrow of mice with **CPh**-induced immunosuppression after treatment with **M-Fuc**, **OS**, **DS** and **r G-CSF**.

The effect of tested compounds on bone marrow cells was further elucidated. After **CPh** treatment, a decrease in the number of progenitor cells CD34^+^ was observed (Figure 2). Injection of **M-Fuc** or **DS** in therapeutic mode did not result in a significant recovery of cells, while administration of **r G-CSF** and **OS** led to a sufficient increase in the cell concentration. Notably, the effect was more pronounced in the latter case.

**Figure 3:**
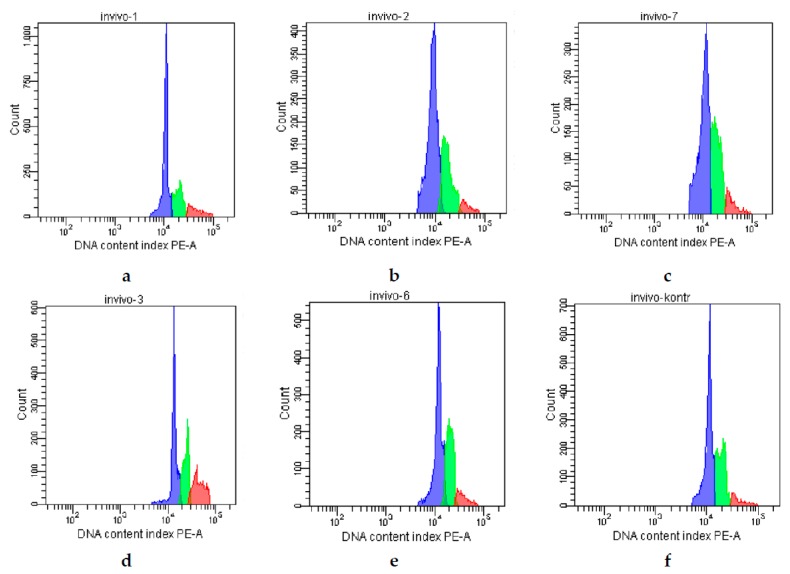
Analysis of the bone marrow cells cycle in mice with **CPh**-induced immunosuppression after treatment by tested compounds: (**a**) positive control—intact mice; (**b**) negative control—**CPh**; (**c**) **CPh** + **M-Fuc**; (**d**) **CPh** + **OS**; (**e**) **CPh** + **DS**; (**f**) **CPh** + **r G-CSF**.

Analysis of the bone marrow cell cycle revealed that an inhibition of the proliferation was observed after **CPh** administration, which resulted in a decrease in the concentration of cells in the mitosis state (G2/M) from 14% to 4% (Figure 3, Table 6). After a course of all tested compounds, an increase in the proliferative index of the cells was detected. Injection of **r G-CSF**, **M-Fuc**, and **DS** resulted in an increase in this value by 2 times; in the **CPh** + **OS** group, the number of cells in G2/M phase reached up to 25%, which was ~3 times higher than that in the **CPh** + **r G-CSF** group.

**Figure 4:**
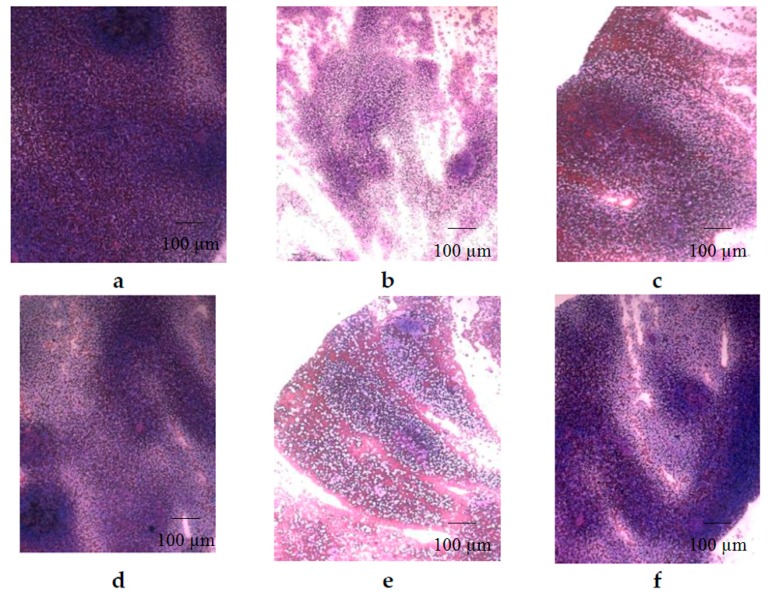
Morphology of the spleen of mice with **CPh**-induced immunosuppression after treatment with tested compounds (hematoxylin-eosin staining): (**a**) positive control—intact mice; (**b**) negative control—**CPh**; (**c**) **CPh** + **M-Fuc**; (**d**) **CPh** + **OS**; (**e**) **CPh** + **DS**; (**f**) **CPh** + **r G-CSF**. Original magnification ×400.

Analysis of the morphology of spleen on smear prints showed that after **CPh** treatment, a myelosuppression was accompanied by a depletion of the cellular composition of the white pulp (Figure 4b). Thus, the follicles (B-dependent areas) and periarteriolar sheaths (T-dependent areas) disappeared. The architecture of the spleen was destroyed, and the interstitial tissue was composed of a dense and uniform layer of lymphoid cells. After a course of **OS** and **r G-CSF**, cell recovery was observed (Figure 4d,f), while **M-Fuc** and **DS** demonstrated a moderate effect (Figure 4c,e).

## 3. Discussion

Proliferation and differentiation of hematopoietic stem/progenitor cells are known to depend on specific local microenvironment formed by bone marrow vascular niches [5,6]. Endothelium of bone marrow is found to express cell adhesion molecules involved in cell signaling by interaction with glycoprotein and glycolipid ligands of HSPCs [6,7,8]. Recently, it was shown that selectins and integrins play a crucial role in regulation of hematopoiesis in experimental animals with myelosuppression after chemotherapy [9,10,20,21,22].

Polysaccharides fucoidans and fucose-enriched structures are traditionally considered as inhibitors of P- and L-selectins but not of E-selectin [23,24,25,26]. The value of the effect depends considerably on the structural features of these molecules [24]. In this study, we have demonstrated that the structure of the studied compounds related to fucoidans significantly influenced the ability to stimulate hematopoiesis in mice with **CPh**-induced myelosuppression. Synthetic octasacharide **OS** was shown to be the most active sample capable of recovering the WBC, RBC, and hemoglobin levels—as well as the absolute number of the neutrophils, monocytes, and lymphocytes—to intact control levels. At the same time, low molecular, weight-modified fucoidan **M-Fuc** with lower degree of sulfation and synthetic disaccharide **DS** did not show any significant effects in the experiments, indicating that the degree of sulfation and MW are important parameters for this type of activity.

Notably, the number of neutrophils in the **CPh** + **OS** group was 2 times higher than that in the **CPh** + **r G-CSF** group (Table 2). Moreover, the phagocytic activity of these cells exceeded that of the granulocytes in the **CPh** + **r G-CSF** group (Table 5).

Analysis of the bone marrow cell cycle revealed that **OS** stimulated the proliferation of hematopoietic progenitor cells ~3 times more effectively than **r G-CSF** (Table 6). The number of these cells in the **CPh** + **OS** group was also ~1.5 times higher than that in the **r G-CSF** group (Figure 2). Additionally, **OS** was shown to effectively stimulate the reparation of spleen structure after **CPh**-induced myelosuppression (Figure 4).

Therefore, totally sulfated synthetic octasaccharide **OS** has been shown to be an effective stimulator of hematopoiesis, with its activity exceeding that of **r G-CSF** regarding the recovery of WBC, RBC, and hemoglobin levels, the number and activity of neutrophils in the blood, and the number of hematopoietic progenitor cells in the bone marrow. These results could be considered as a base for the development of a drug for the treatment and prevention of immunosuppression complications. In addition, **OS** derivatives could be applied for the construction of hybrid systems [27] with more potent biological effects. 

## 4. Materials and Methods

### 4.1. General Methods

Immunophenotype and membrane-associated markers on blood and bone marrow cells were examined using anti-mouse antibodies CD11c, CD62p, CD34, CD11c, CD3, CD4, CD8, and NK1.1 (Becton Dickinson Bioscience, San Jose, CA, USA). The phagocytic activity was studied using the FagoFlow Ex Kit (Exbio, Praha, Czech Republic). BD Canto II flow cytometer (Becton Dickinson Bioscience, San Jose, CA, USA) was used for the study. Sample preparation was carried out in accordance with the manufacturer’s instructions. All measurements were carried out in triplets. To evaluate each parameter, the blood of 4 mice of each group was used. Cell cycle analysis was performed on Muse Cell Analyzer (Merck KGaA, Darmstadt, Germany) using the Muse Cell Cycle Kit (EMD Millipore Corporation, Billerica, MA, USA).

### 4.2. Sulfated Polysaccharides

Modified fucoidan **M-Fuc** was prepared by chemical modification of fucoidan from *C. flageliformis* as described previously [14]. Octasaccharide **OS** [15,16] and disaccharide **DS** [17,18] were synthesized from l-fucose.

### 4.3. Animal Model

The animal protocols used in this work were evaluated and approved by the local ethical committee of the N.N. Blokhin National Medical Research Center of Oncology (Protocol 12-2017). They are in accordance with the order 490 (5 November 2008) of the Agricultural Ministry of Russian Federation and meet National GLP Standard of Russian Federation (53434-2009) and European Convention for the Protection of Vertebrate Animals used for Experimental and Other Scientific Purposes (Strasbourg, France, 18.03.1986).

Thirty-six mice of the Balb/c line (males, weight 20 ± 2 g) were divided into 6 groups with 6 animals in each group. Before and during the experiment, the animals were in standardized vivarium conditions (at 20 ± 2 °C with free access to food and water). For the inducing of myelosuppression, **CPh** (Endoxan, Baxter, Germany) in a dosage of 100 mg/kg was injected to animals of 5 groups once daily intraperitoneally for 4 days. Then, the following sterile solutions (0.2 mL) were administered subcutaneously to all animals for 3 days (1 time daily): 0.5 mg/mL of **M-Fuc** in isotonic sodium chloride solution (**CPh** + **M-Fuc** group), 0.5 mg/mL of **OS** in isotonic sodium chloride solution (**CPh** + **OS** group), 0.5 mg/mL of **DS** in isotonic sodium chloride solution (**CPh** + **DS** group), and 3 nmol/mL of **r G-CSF** (Leucita, Sygardis AqVida, Germany) in isotonic sodium chloride solution (**CPh** + **r G-CSF** group), sterile isotonic sodium chloride solution (**CPh** group). A sterile isotonic sodium chloride solution was administered to the mice of the control group in the same regime. The animals were euthanized by decapitation after 2 days. Blood of each animal was collected in the tubes with ethylenediaminetetraacetic acid (EDTA), the spleen was removed from the animals, and smears were imprinted on the polyethylene-coated glasses (Gerhard Menzei GmbH, Termo Scientific, Braunschweig, Germany). The fingerprints were fixed in May-Grunwald solution, stained with hematoxylin-eosin (HE) and analyzed by light microscopy. Hematologic parameters of blood were analyzed on an automatic analyzer, determining the concentration of WBC, platelets, and RBC. Bone marrow cells were isolated from the femurs.

### 4.4. Statistical Analysis

Data in the group were presented in the format of mean and standard deviation (Mean ± SD). An analysis of the reliability of the differences was carried out using the *t* criterion. Differences were considered significant at *p* < 0.05.

## 5. Conclusions

Modified polysaccharide **M-Fuc** prepared from the fucoidan from the seaweed *Chordaria flagelliformis*, along with related synthetic octasaccharide **OS** and disaccharide **DS**, were studied as stimulators of hematopoiesis on a model of cyclophosphamide immunosuppression in mice. Recombinant granulocyte colony-stimulating factor (**r G-CSF**), which is currently applied in medicine to treat low blood neutrophils, was used as a reference. Polysaccharide **M-Fuc** and sulfated difucoside **DS** did not demonstrate significant effect in the experiments, while related sulfated octasaccharide **OS** was shown to be an effective stimulator of hematopoiesis, with its activity exceeding that of **r G-CSF** regarding the recovery of WBC, RBC, and hemoglobin levels, the number and activity of neutrophils in blood, and the number of hematopoietic progenitor cells in the bone marrow. These results could be considered as a base for development of a drug for treatment and prevention of immunosuppression complications.

## Figures and Tables

**Table 1:** Hematologic parameters of mice with cyclophosphamide (**CPh**)-induced immunosuppression after treatment with modified fucoidan **M-Fuc**, octasaccharide **OS**, disaccharide **DS**, and recombinant granulocyte colony-stimulating factor (**r G-CSF**) (Mean ± SD).

Groups	WBC (×10^3^/µL)	RBC (×10^6^/µL)	Hemoglobin (g/dL)	Platelets (×10^3^/µL)
Control	6.7 ± 1.14	9.4 ± 0.45	16.6 ± 0.64	571.5 ± 54.71
**CPh**	2.4 ± 0.36	5.4 ± 0.07	11.3 ± 1.73	812.3 ± 46.35
**CPh** + **M-Fuc**	1.9 ± 1.20	5.4 ± 2.95	10.2 ± 5.52	589.0 ± 350.06
**CPh** + **OS**	5.3 ± 0.93	9.1 ± 1.11	16.7 ± 2.33	1036.3 ± 104.29
**CPh** + **DS**	2.3 ± 1.82	4.7 ± 2.89	8.7 ± 5.47	389.6 ± 207.74
**CPh** + **r G-CSF**	3.7 ± 0.47	7.0 ± 0.77	13.0 ± 1.30	541.0 ± 44.20

**Table 2:** Different populations of leucocytes in blood of mice with **CPh**-induced immunosuppression after treatment with **M-Fuc**, **OS**, **DS**, and **r G-CSF** (Mean ± SD).

Groups	Neutrophils (×10^3^/µL)	Monocytes (×10^3^/µL)	Lymphocytes (×10^3^/µL)
Control	1.9 ± 0.21	0.5 ± 0.01	4.4 ± 1.32
**CPh**	0.4 ± 0.25	0.2 ± 0.12	1.5 ± 0.38
**CPh** + **M-Fuc**	0.5 ± 0.39	0.2 ± 0.10	1.2 ± 0.71
**CPh** + **OS**	2.1 ± 0.88	0.5 ± 0.22	2.3 ± 0.02
**CPh** + **DS**	1.3 ± 1.22	0.1 ± 0.01	0.6 ± 0.32
**CPh** + **r G-CSF**	1.1 ± 0.74	0.4 ± 0.09	2.1 ± 0.47

**Table 3:** Different subpopulations of lymphocytes in blood of mice with **CPh**-induced immunosuppression after treatment with **M-Fuc**, **OS**, **DS**, and **r G-CSF**.

Groups	CD3^+^CD4^+^ (%)	CD3^+^CD8^+^ (%)	The Ratio CD4^+^/CD8^+^	NK (%)
Control	31 ± 0.7	16 ± 3.8	1.94 ± 0.28	1.3 ± 0.8
**CPh**	14 ± 3.4	16 ± 3.1	0.88 ± 0.10	2.1 ± 2.0
**CPh** + **M-Fuc**	16 ± 3.0	15 ± 2.2	1.07 ± 0.10	0.4 ± 1.2
**CPh** + **OS**	29 ± 2.4	15 ± 4.1	1.93 ± 0.19	2.3 ± 1.1
**CPh** + **DS**	24 ± 2.8	17 ± 1.4	1.41 ± 0.07	1.3 ± 0.2
**CPh** + **r G-CSF**	22 ± 0.8	16 ± 2.1	1.38 ± 0.50	3.2 ± 1.1

**Table 4:** Expression of CD11c and CD62p on the granulocytes of mice with **CPh**-induced immunosuppression after treatment with **M-Fuc**, **OS**, **DS** and **r G-CSF**.

Groups	CD11c^+^		CD62p^+^	
	%	*p*	%	*p*
Control	44 ± 5.4	-	48 ± 2.7	-
**CPh**	68 ± 8.1	0.057	70 ± 6.8	0.029
**CPh** + **M-Fuc**	27 ± 3.1	0.047	8 ± 2.4	0.001
**CPh** + **OS**	34 ± 6.2	0.278	38 ± 4.1	0.097
**CPh** + **DS**	22 ± 3.9	0.021	23 ± 2.5	0.002
**CPh** + **r G-CSF**	13 ± 7.5	0.02	14 ± 8.4	0.012

**Table 5:** The effect of the tested compounds on the functional activity of the blood granulocytes in mice with **CPh**-induced immunosuppression.

Groups	*E. coli*^+^ Cells		ROS^+^ Cells	
	%	*p*	%	*p*
Control	32 ± 3.4	-	31 ± 1.6	-
**CPh**	54 ± 4.1	0.009	36 ± 0.1	0.026
**CPh** + **M-Fuc**	57 ± 3.1	0.003	35 ± 1.4	0.119
**CPh** + **OS**	43 ± 6.2	0.181	29 ± 1.1	0.350
**CPh** + **DS**	52 ± 3.9	0.012	47 ± 1.8	0.001
**CPh** + **r G-CSF**	29 ± 3.5	0.056	51 ± 0.8	0.001

**Table 6:** Different phases of the bone marrow cells of mice with **CPh**-induced immunosuppression after treatment with **M-Fuc**, **OS**, **DS**, and **r G-CSF**.

Groups	G0/G1 (%)	S (%)	G2/M (%)
Control	63	23	14
**CPh**	65	29	4
**CPh** + **M-Fuc**	68	24	8
**CPh** + **OS**	47	28	25
**CPh** + **DS**	58	34	8
**CPh** + **r G-CSF**	60	32	8
